# *MYC* amplifications are common events in childhood osteosarcoma

**DOI:** 10.1002/cjp2.219

**Published:** 2021-05-09

**Authors:** Solange De Noon, Jannat Ijaz, Tim HH Coorens, Fernanda Amary, Hongtao Ye, Anna Strobl, Iben Lyskjær, Adrienne M Flanagan, Sam Behjati

**Affiliations:** ^1^ Research Department of Pathology University College London (UCL) Cancer Institute London UK; ^2^ Wellcome Sanger Institute Hinxton UK; ^3^ Department of Histopathology Royal National Orthopaedic Hospital Stanmore UK; ^4^ Medical Genomics Research Group UCL Cancer Institute London UK; ^5^ Cambridge University Hospitals NHS Foundation Trust Cambridge UK; ^6^ Department of Paediatrics University of Cambridge Cambridge UK

**Keywords:** *MYC*, *CCNE1*, osteosarcoma, genomics, copy number variants

## Abstract

Osteosarcoma, the most common primary malignant tumour of bone, affects both children and adults. No fundamental biological differences between paediatric and adult osteosarcoma are known. Here, we apply multi‐region whole‐genome sequencing to an index case of a 4‐year‐old child whose aggressive tumour harboured high‐level, focal amplifications of *MYC* and *CCNE1* connected by translocations. We reanalysed copy number readouts of 258 cases of high‐grade osteosarcoma from three different cohorts and identified a significant enrichment of focal *MYC*, but not *CCNE1*, amplifications in children. Furthermore, we identified four additional cases of *MYC* and *CCNE1* coamplification, highlighting a rare driver event which warrants further investigation. Our findings indicate that amplification of the *MYC* oncogene is a major driver of childhood osteosarcoma, while *CCNE1* appears recurrently amplified independent of age.

## Introduction

Osteosarcoma is the most common primary cancer of bone, typically arising in long bones during periods of rapid bone growth in puberty, with a second peak in old age often associated with radiation and Paget's disease. The prognosis of osteosarcoma does not vary significantly across the age range, although some studies have demonstrated poorer outcomes in adolescence compared with younger patients [1, 2, 3]. These differences may be accounted for by the tolerability of systemic cytotoxic chemotherapy, which, for example, is thought to be reduced in adolescents compared to young children [1]. Over the past decade, studies have shown that there are no fundamental differences in the osteosarcoma genome of children (<18 years) and adults [4, 5].

The outlook of osteosarcoma has not substantially improved since the addition of multiagent cytotoxic chemotherapy to treatment protocols in the early 1980s [6]. With current approaches, the 5‐year survival stands at 60–70% in those presenting without metastatic disease [7]. Predictors of poor outcome include metastatic disease at presentation, incomplete resection, and poor histological response to neoadjuvant chemotherapy [7]. Apart from the amplification of specific oncogenes including *FGFR1* [8] and *MYC* [9, 10, 11] that have been associated with poor clinical outcomes, there has been limited success in identifying potential prognostic biomarkers for osteosarcoma.

Based on an index case of a young child whose aggressive disease was driven by coamplification of *CCNE1* and *MYC*, we investigated the prevalence of amplification and coamplification of these two genes in multiple osteosarcoma data sets, and whether these genes herald a particularly aggressive disease course.

## Materials and methods

### Genomic analysis of index case

**Figure 1:**
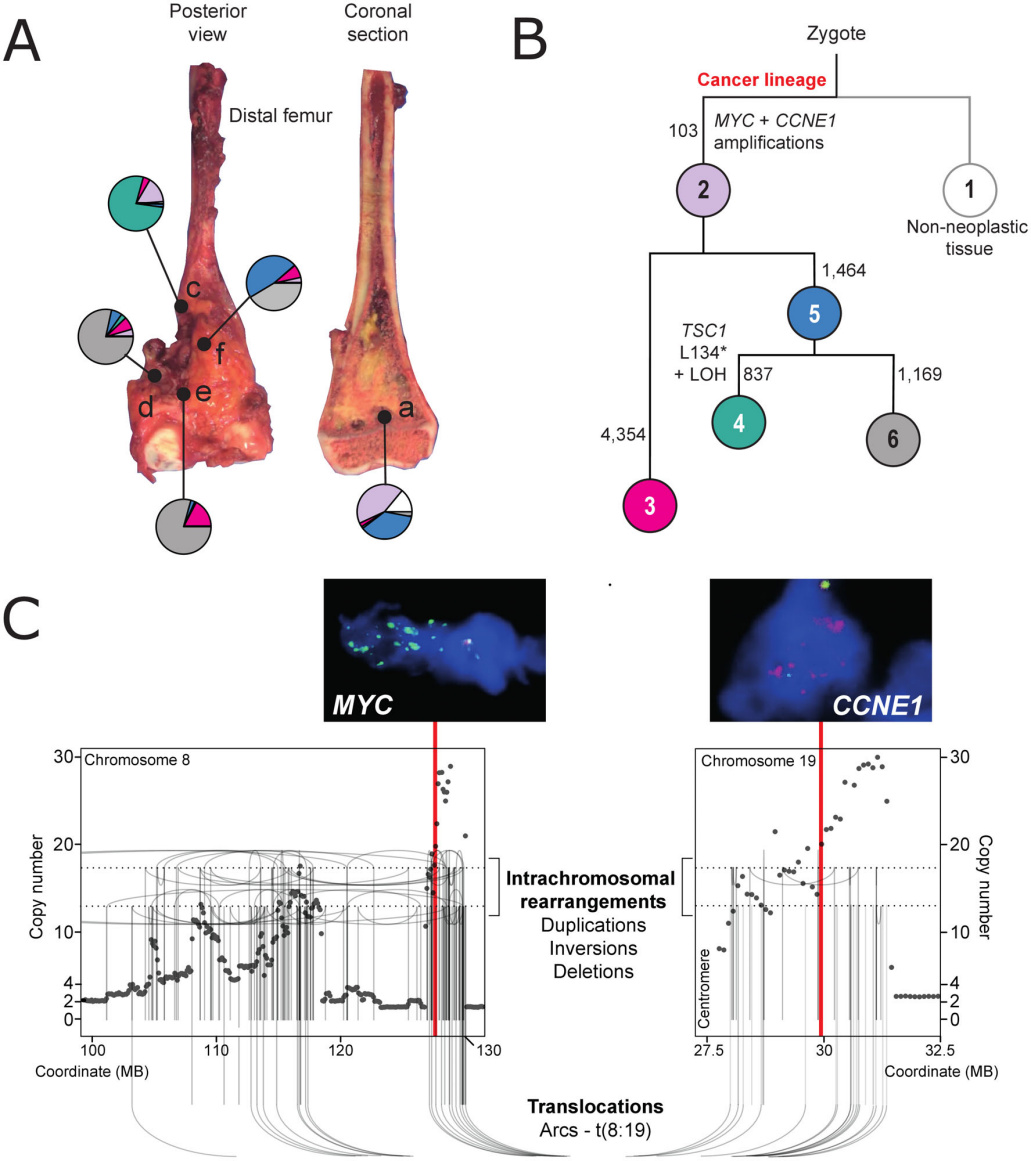
Multi‐sample whole‐genome analysis of the index case. (A) Image of resected tumour highlighting sampled tumour regions with pie charts (colouring as per B) demonstrating the relative proportion of clones comprising each region, as identified through phylogenetic analysis (Canopy algorithm). Letters refer to sample IDs. (B) Tumour phylogeny. Each circle represents a clone (number in circle = clone ID). The number next to each branch represents branch defining substitutions. The lengths of branches represent these only qualitatively. Driver events are highlighted next to each branch. (C) Consensus rearrangements and copy number across tumour samples with corresponding FISH validation. Vertical red lines highlight the position of *MYC* and *CCNE1*. Each dot represents the copy number (*y*‐axis) of a specific genomic coordinate (*x*‐axis). Lines and arc show the position and relationship of breakpoints.

We subjected five samples of the resection specimen (Figure 1A) and two blood samples from the index case to whole‐genome sequencing (coverage ranging 32–102×), as previously described [4] (see supplementary material, Supplementary materials and methods). Sequencing reads were analysed using the extensively validated analysis pipeline of the Wellcome Sanger Institute, to call substitutions (Caveman algorithm [12]), indels (Pindel [13]), and rearrangements (BRASS [14]). The clonal composition of tissues was determined using the Canopy algorithm [15] (see supplementary material, Table S1). The patient's legal guardian provided informed consent for participation in this study and ethical approval for this study was obtained from the Cambridge Central Research Ethics Committee (National NHS Research Ethics Service [reference 16/EE/0394]).

### Analysis of copy number data

We reanalysed array‐based copy number readouts of high‐grade osteosarcomas from three cohorts: International Cancer Genome Consortium (ICGC) data set (*n* = 54; segment files provided in publication [4]; Affymetrix Genome‐Wide Human SNP Array 6.0 (Applied Biosystems, Foster City, CA, USA) and *in silico* arrays derived from whole‐genome sequences), German‐Swiss published data set [16] (*n* = 124; Affymetrix Cytoscan HD array (Applied Biosystems, Foster City, CA, USA) raw data files [ArrayExpress accession E‐MTAB‐4815]; segment files generated with ASCAT algorithm [17]), and Therapeutically Applicable Research to Generate Effective Treatments (TARGET) Osteosarcoma data set (*n* = 80; publicly available segment files [dbGaP Sub‐study ID phs000468] generated from Affymetrix Human SNP Array 6.0 data). We applied a widely used definition [4] of focal amplifications, i.e. a total copy number to tumour ploidy ratio of over 2, and an amplicon that was smaller than 2 Mb.

### Analysis of *MYC* and *CCNE1* copy number by FISH

Twenty‐three cases of paediatric osteosarcoma diagnosed at the Royal National Orthopaedic Hospital (RNOH) between 2011 and 2018 were subjected to fluorescence in situ hybridization (FISH) analysis of *MYC* and *CCNE1* as described previously (see supplementary material, Supplementary materials and methods) [18]. Probes used for *MYC* and *CCNE1* were MYC/CEN8 dual colour probe (Zytovision, Bremerhaven, Germany) and CCNE1/CEN19p dual colour probe (Abnova, Taipei, Taiwan), respectively.

### Statistical analyses

Binomial regression analysis was performed to measure the association between patient age and our determined *MYC* and *CCNE1* copy states using the ‘glm’ function of the R stats package. Survival analysis was performed using the Kaplan–Meier method and survival curves were plotted using the ‘survival’ and ‘survminer’ R packages.

## Results and discussion

The impetus for this study was the case of a 4‐year‐old child treated for osteosarcoma of the distal femur with an unusually aggressive disease course. At the time of diagnosis, she had a rapidly growing primary tumour with a skip lesion in the affected bone and lung metastases. She underwent neoadjuvant MAP chemotherapy (cisplatin, methotrexate, and doxorubicin) followed by surgical resection and two cycles of post‐operative MAP chemotherapy, as per the European and American Osteosarcoma study group (EURAMOS) protocol [19]. She developed bulky metastatic lung disease within 8 weeks of cessation of treatment, to which she succumbed. Histological examination of the resection specimen revealed extensive viable high‐grade osteosarcoma, from which we subjected five samples to whole‐genome sequencing (Figure 1A). Analysis of all classes of somatic variants revealed typical somatic features of osteosarcoma, including complex structural variants [4, 20] (Figure 1B,C and supplementary material, Table S2). The clonal composition of different tumour regions showed spatial diversity (Figure 1A,B and supplementary material, Table S1). Two somatic driver events were common to all tumour regions – high‐level gains of both *MYC* and *CCNE1* (Figure 1C). We determined that these amplifications were generated by a single chromothripsis‐driven amplification event on chromosomes 8q (*MYC*) and 19q (*CCNE1*), interconnected by multiple translocations (Figure 1C and supplementary material, Figure S1). Chromothripsis‐driven amplification is a recognised mechanism of oncogene amplification in a subset of tumours across several cancer types [21], and has been previously described as a recurrent event in osteosarcoma [4].

### *MYC* amplifications are enriched in childhood osteosarcoma

We next reanalysed copy segment data from our previously published data set of 54 high‐grade osteosarcoma exomes and genomes (ICGC cohort [4]). Focal amplifications of *MYC* and *CCNE1* were detected in 15 and 13% of the ICGC cohort, respectively. These events were particularly common in children, with 40 and 20% of patients under the age of 13 (*n* = 15) harbouring *MYC* and *CCNE1* amplifications, respectively, compared with 5% (*p* = 0.002, chi‐square test) and 10% (*p* = 0.6, chi‐square test) in the older population (*n* = 39). Looking at driver alterations across the cohort, only *TERT* mutations showed a notable difference in frequency between *MYC*‐amplified and non‐amplified cases; however, this was not statistically significant after multiple hypothesis testing (false discovery rate (FDR) adjusted *P* value: 0.54).

**Figure 2:**
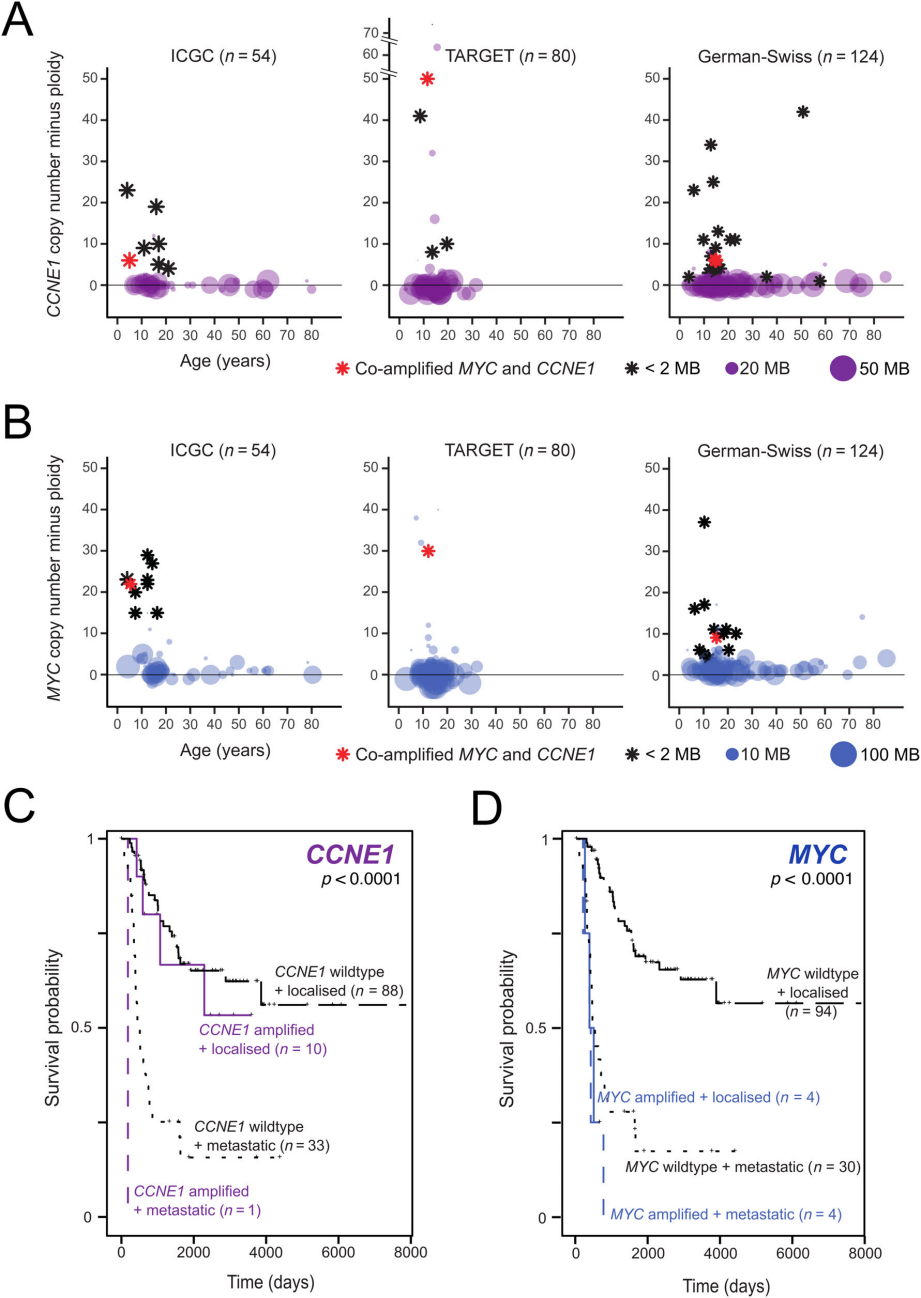
*MYC* and *CCNE1* amplifications in osteosarcoma. Genomic segments harbouring (A) *CCNE1* or (B) *MYC* are shown across three independent cohorts. In each plot, circles or stars represent data from an individual tumour. The position on the *x*‐axis shows the age. The *y*‐axis position represents absolute gene copy number minus tumour ploidy. The sizes of circles represent the segment size with focal amplifications (total copy number to ploidy ratio > 2 and amplicon size ≤2 Mb) represented by black stars. Red stars identify tumours with coamplification of *MYC* and *CCNE1*. Effects of (C) *CCNE1* or (D) *MYC* amplification on survival (Kaplan–Meier analysis) by metastatic status. Survival analysis performed on ICGC and TARGET‐OS cohorts only.

We then analysed copy number data of osteosarcomas from two publicly available osteosarcoma data sets: the US TARGET Osteosarcoma cohort (TARGET OS, *n* = 80) and a German‐Swiss cohort [16] (*n* = 124). In these, *MYC* was amplified in 1 and 10%, and *CCNE1* was amplified in 5 and 13%, respectively. Statistical analysis performed on all three data sets combined confirmed a significant association between young age and the presence of *MYC* amplifications (*p* < 0.02, binomial model, see *Materials and methods*), but not *CCNE1* gains (*p* > 0.05). Next, we examined the age distribution, copy number, and size of genomic segments harbouring *MYC* or *CCNE1* across the three cohorts and observed that they shared a consistent pattern of high‐level, focal copy number gains of *MYC* and *CCNE1* in patients younger than 18 years (Figure 2A,B). Finally, we employed FISH in a fourth tumour collection from 23 anonymised paediatric osteosarcomas assembled from the archives of the Pathology Department of the RNOH (Stanmore, UK). Informative readouts were obtained from 22 out of 23 cases (see supplementary material, Table S3). Of these, one tumour exhibited coamplification of *MYC* and *CCNE1*, five cases amplification of *CCNE1* alone, and another five cases *MYC* amplification, corroborating the genomic finding of these alterations in paediatric osteosarcoma.

Survival analysis jointly performed on the ICGC and TARGET OS data sets demonstrated an association between *MYC* amplification and inferior outcomes (Figure 2C) confirming previous reports. In contrast, *CCNE1* amplification did not correlate with adverse survival (Figure 2D). No significant association was found between survival and amplification of either gene in our FISH cohort (see supplementary material, Figure S2), possibly as these results were not subject to the focal amplification criteria applied to our copy number data.

### Coamplification of *MYC* and *CCNE1* is a rare event in osteosarcoma

Four further tumours demonstrating gains in both *MYC* and *CCNE1* were identified in addition to the index case in patients aged 5–15 years. Each of the three copy number data sets included a single case with this coamplification (Figure 2A,B and supplementary material, Tables S4 and S5). Clinical outcome data were available for two of these cases (PD9980a and PAMLKS) and both demonstrated aggressive behaviour with early metastatic disease and subsequent death. The low frequency of these events suggests that these are rare events possibly occurring as part of the complex structural alterations which characterise osteosarcoma. A case harbouring both *MYC* and *CCNE1* amplifications was also identified in our FISH cohort (R010), which arose in a 8‐year‐old with no adverse events on follow‐up (see supplementary material, Table S3). Greater numbers and detailed analysis of genomic data are therefore required to evaluate this finding fully.

*CCNE1* is a cell cycle regulator controlling G1/S transition, and plays a role in tumourigenesis by contributing to cell proliferation and chromosomal instability [22]. *CCNE1* is a recognised driver in many malignancies including osteosarcoma [16, 23], and has proven to be an independent prognostic factor in both triple‐negative breast cancer and ovarian cancer [24, 25, 26]. As part of the many *MYC*‐regulated pathways which contribute to tumourigenesis, *MYC* has been shown to directly regulate *CCNE1* gene expression as well as CCNE1‐CDK2 complex activity [27, 28], and mouse models have demonstrated the co‐operative action of *MYC* and *CCNE1* in contributing to tumour formation in hepatocellular cancer [29]. However, while recurrent coamplification of *MYC* with other driver genes including *ERRB2* and *EGFR* has previously been reported [30, 31], and in breast cancer is associated with a poorer prognosis [30], coamplification with *CCNE1* has not been previously described.

In this analysis of oncogenic *MYC* and *CCNE1* amplifications in osteosarcoma, we identified a somatic genomic feature that appears to be enriched in paediatric cases. Previous investigations of the osteosarcoma genome have not revealed differences between age groups, bar the expected excess of age‐related base substitutions associated with age, and the greater contribution of pathogenic germline variants to childhood tumours. Furthermore, we report the rare occurrence of coamplification of the known oncogenic drivers *MYC* and *CCNE1*, a finding which warrants further studies. We also provide additional evidence for the utility of *MYC* alterations as a prognostic factor in osteosarcoma.

## Author contributions statement

JI performed sequence analysis of the index case. SDN performed copy number profiling of tumour data sets and clinical data collection. THHC and IL contributed to bioinformatic analyses. HY, AS and FA performed FISH analysis. SDN and JI wrote the manuscript, with contributions from AMF and SB. AMF and SB were responsible for study design and direction of research.

## Supporting information


Supplementary materials and methods

**Figure S1.** Circos plots showing structural rearrangements detected in sequenced distinct regions from the index tumour
**Figure S2.** Survival analysis showing the impact of (A) *MYC* and (B) *CCNE1* amplifications as detected by FISH in the RNOH cohort (*n* = 22)

**Table S1.** Somatic alterations for phylogenetic analysis (Canopy)
**Table S2.** Index case BRASS detected structural rearrangements
**Table S3.***MYC* and *CCNE1* FISH results (RNOH cohort)
**Table S4.***MYC* and *CCNE1* copy number profiles for published data sets
**Table S5.** Survival data, ICGC cohort

## Data Availability

The authors declare that all data supporting the findings of this study are available within the article and its supplementary information files or from the corresponding author on reasonable request. Sequencing data generated by this study are available from the EGA (EGAD00001006859). Previously published sequencing data that support the findings of this study are available from the EGA: EGAD00001000107, EGAS00001000196, and EGAD00001000147 (DOI: 10.1038/ncomms15936), and ArrayExpress: E‐MTAB‐4815 (DOI: 10.1002/ijc.30778). The data used for the TARGET OS data set are available at https://portal.gdc.cancer.gov/projects.
