# Supplementary material for: MYC amplifications are common events in childhood osteosarcoma
Source: J Pathol Clin Res. 2021 May 9;7(5):425–31. doi: 10.1002/cjp2.219 (PMC8363928; doi:10.1002/cjp2.219)
Supplement: Supplementary file 1 — Supplementary materials and methods [file CJP2-7-425-s003.docx]

***MYC* amplifications are common events in childhood osteosarcoma.**

S De Noon *et al*, *J Pathol Clin Res,* DOI 10.1002/cjp2.219

**Supplementary Materials and Methods**

Reference numbers refer to the list in the main paper

**Whole genome sequencing**

DNA was extracted from blood or fresh frozen tumour samples using Qiagen DNeasy kit (Qiagen, FRITSCH GmbH, Idar-Oberstein, Germany), following the manufacturer’s instructions*.* Short insert 500bp genomic libraries were constructed and used to prepare flow cells and generate clusters using standard Illumina no-PCR library protocols The Illumina HiSeq X platform (Illumina Cambridge, Ltd, Little Chesterford, UK) was used to generate a paired-end (150bp) whole genome sequencing with coverage varying from 32X-102X.

**DNA sequence alignment**

Alignment to human reference genome GRCh37 was done using the Burrows-Wheeler algorithm (BWA-Mem).

**Variant detection**

The Cancer Aging and Somatic Mutation (Welcome Sanger Institute) pipelines were used to call variants. Single nucleotide variants were called using the CaVEMan algorithm [12] and were filtered on PASS variants, ASMD greater than or equal to 140 and CLPM equal to 0. Indels were called using Pindel [13]. Copy number variants were called using ASCAT [17]. Structural rearrangements were called using BRASS [14] and validated by the BRASS implementation of local assembly.

**Tumour phylogeny**

Mutations were clustered based on the variant allele fraction of the single nucleotide variants in tumour samples and these clusters were subsequently used to generate a phylogenetic tree of subclones within the tumour. This was done by the Canopy algorithm [15]. Structural variants were added manually to the phylogeny using copy number changes and variant allele fractions.

**Circos plots**

Circos plots were generated using the circos algorithm.

**Copy number profiling of published datasets**

*ICGC cohort*

Tumour ploidy and copy number analysis of 54 high grade conventional osteosarcomas were derived from whole genome sequences (n=4) or Affymetrix SNP 6.0 arrays (n= 50) was performed using ascatNgs or ASCAT algorithms respectively, as part of a previous publication [4] and segmentation files retrieved for analysis. Additional driver alteration calls as originally defined were also utilised.

*TARGET Osteosarcoma cohort*

Copy number segmentation data and tumour ploidy (generated from Affymetrix SNP 6.0 arrays using CNVkit algorithm with ploidy correction by PureCN algorithm), as well as clinical annotations for 88 osteosarcomas were retrieved from the U.S. National Cancer Institute Office of Cancer Genomics data portal under dbGaP Sub-study ID phs000468 available at <https://ocg.cancer.gov/programs/target/data-matrix> and [https://portal.gdc.cancer.gov/projects.](https://portal.gdc.cancer.gov/projects) Two cases without accompanying ploidy data and 6 cases with breakpoints identified within the *CCNE1* gene were excluded from the analysis.

*German-Swiss cohort*

Affymetrix Cytoscan HD array data for 160 previously published osteosarcomas were retrieved from ArrayExpress under accession E-MTAB-4815 [16]. LogR and BAF were generated from raw CEL files using ‘Rawcopy’ R package and copy number segmentation performed with ASCAT. Thirty-six cases failed ASCAT (insufficient tumour purity or inability to find optimal ploidy and cellularity) and were excluded from the analysis.

*MYC and CCNE1 copy state determination*

For each of the three datasets, segments harbouring *MYC* and CCNE1 genes were identified, and genes were considered focal amplifications based on two criteria: (i) segment total copy number divided by tumour ploidy was greater than 2 and (ii) segment size (segment end position - start position) was less than 2 megabases. Tumours which harboured breakpoints within either gene were excluded from further analysis.

**Statistical analysis**

Binomial regression analysis was performed to measure the association between patient age and the presence of *MYC* and *CCNE1* amplifications using the ‘glm’ function (family = “binomial”) of the R Stats package. Overall survival for the ICGC and TARGET OS datasets was analysed by the Kaplan Meier method and survival curves plotted using the ‘survival’ and ‘survminer’ R packages respectively.

**FISH analysis**

Thirty paediatric osteosarcomas (patients aged 12 and under) with primary resection at the Royal National Orthopaedic Hospital in Stanmore were selected for FISH analysis, with material from 23 cases suitable for study. Commercially available probes were used for this study: *MYC/CEN8* dual colour probe (Zytovision, Bremerhaven, Germany) and *CCNE1/CEN19p* dual colour probe (Abnova, Taipei, Taiwan). FISH was performed on paraffin sections as described previously [18]. A minimum of 50 consecutive non-overlapping nuclei were counted. A result was considered positive when the ratio of target gene to centromeric/telomeric control region was >2 in at least 10% of nuclei.
