# Supplementary material for: MYC amplifications are common events in childhood osteosarcoma
Source: J Pathol Clin Res. 2021 May 9;7(5):425–31. doi: 10.1002/cjp2.219 (PMC8363928; doi:10.1002/cjp2.219)
Supplement: Supplementary file 2 — Figure S1. Circos plots showing structural rearrangements detected in sequenced distinct regions from the index tumour Figure S2. Survival analysis showing the impact of (A) MYC and (B) CCNE1 amplifications as detected by FISH in the RNOH cohort (n = 22) [file CJP2-7-425-s002.docx]

***MYC* amplifications are common events in childhood osteosarcoma**

S De Noon *et al*, *J Pathol Clin Res,* DOI 10.1002/cjp2.219

**Supplementary Figures**

**Figure S1.** Circos plots (A-E) showing structural rearrangements detected in sequenced distinct regions (PD40312a, PD40312c, PD40312d, PD40312e, PD40312f) from the index tumour. Black lines indicate interchromosomal translocations, blue lines are intrachromosomal rearrangements and red lines show interchromosomal rearrangements involving chr8 and chr19.


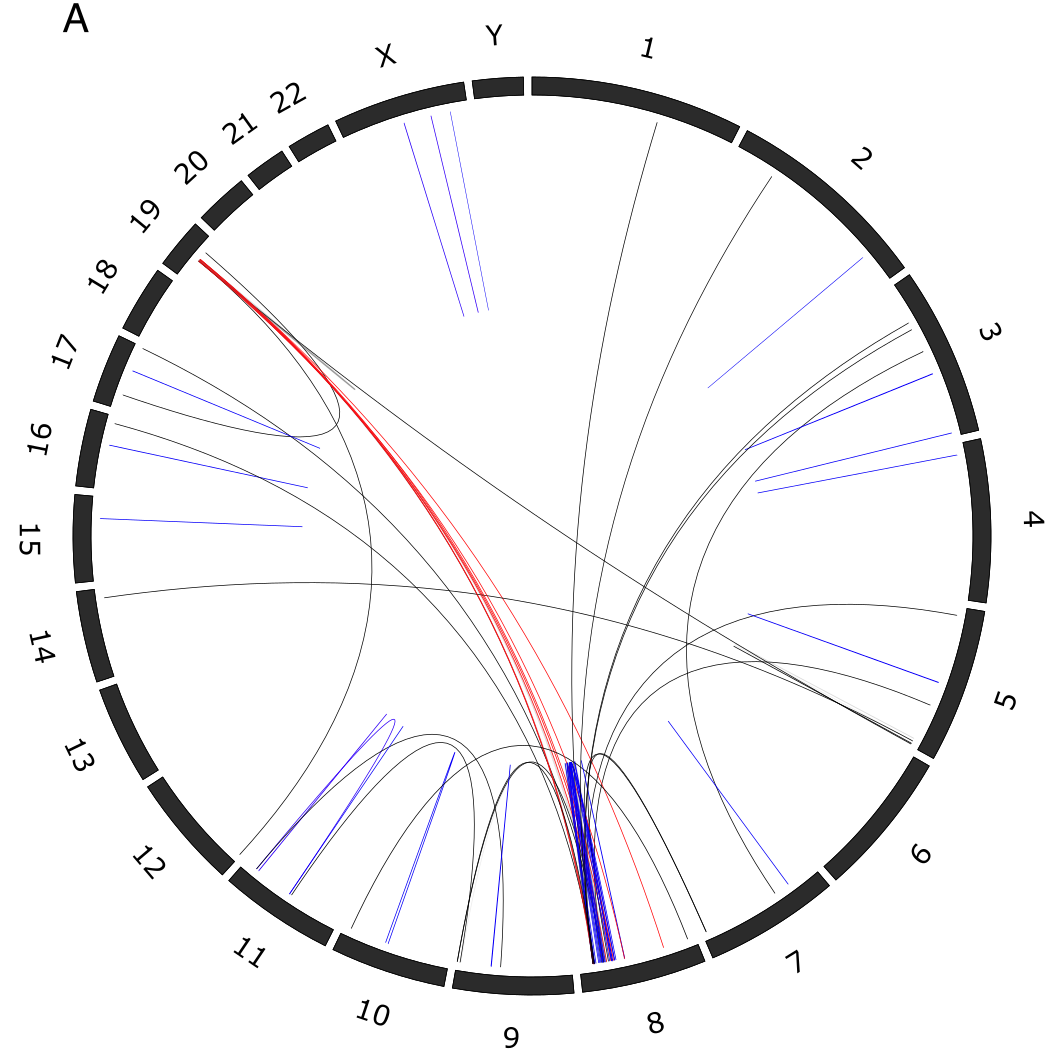


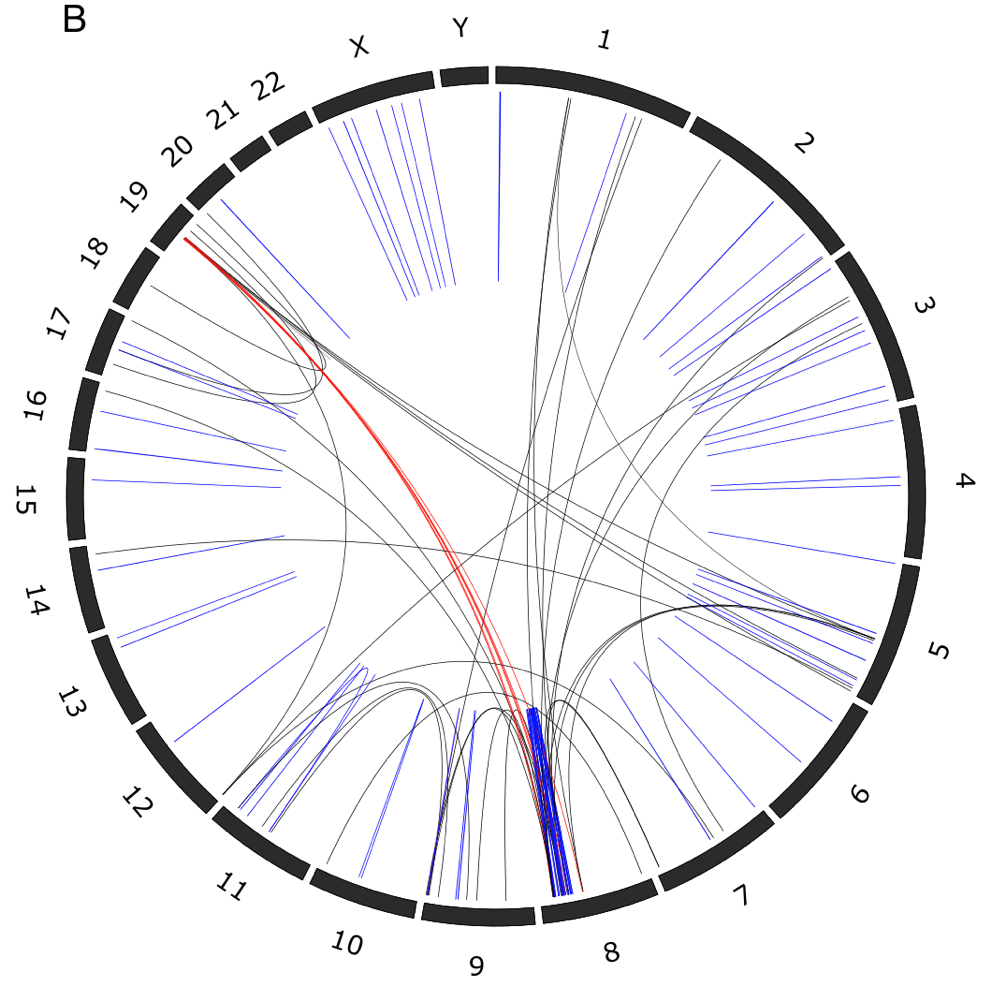

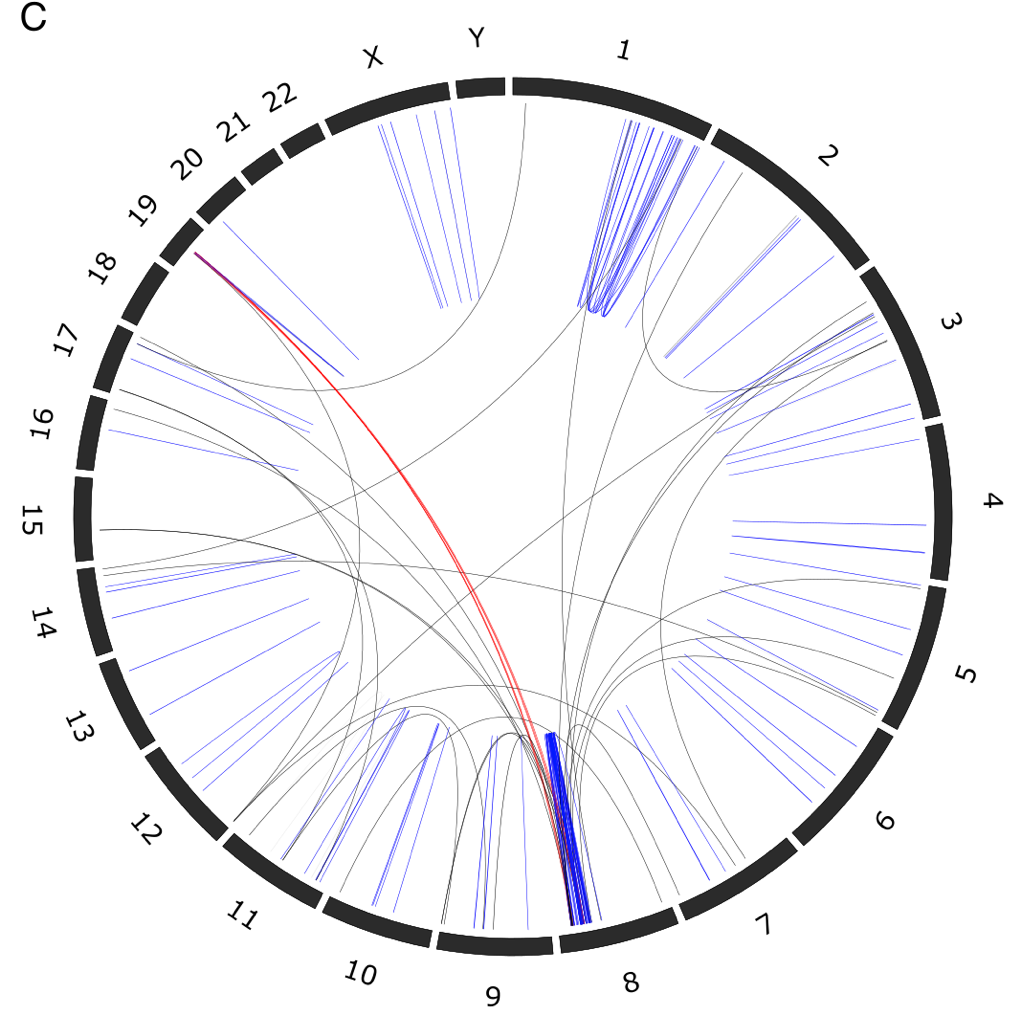


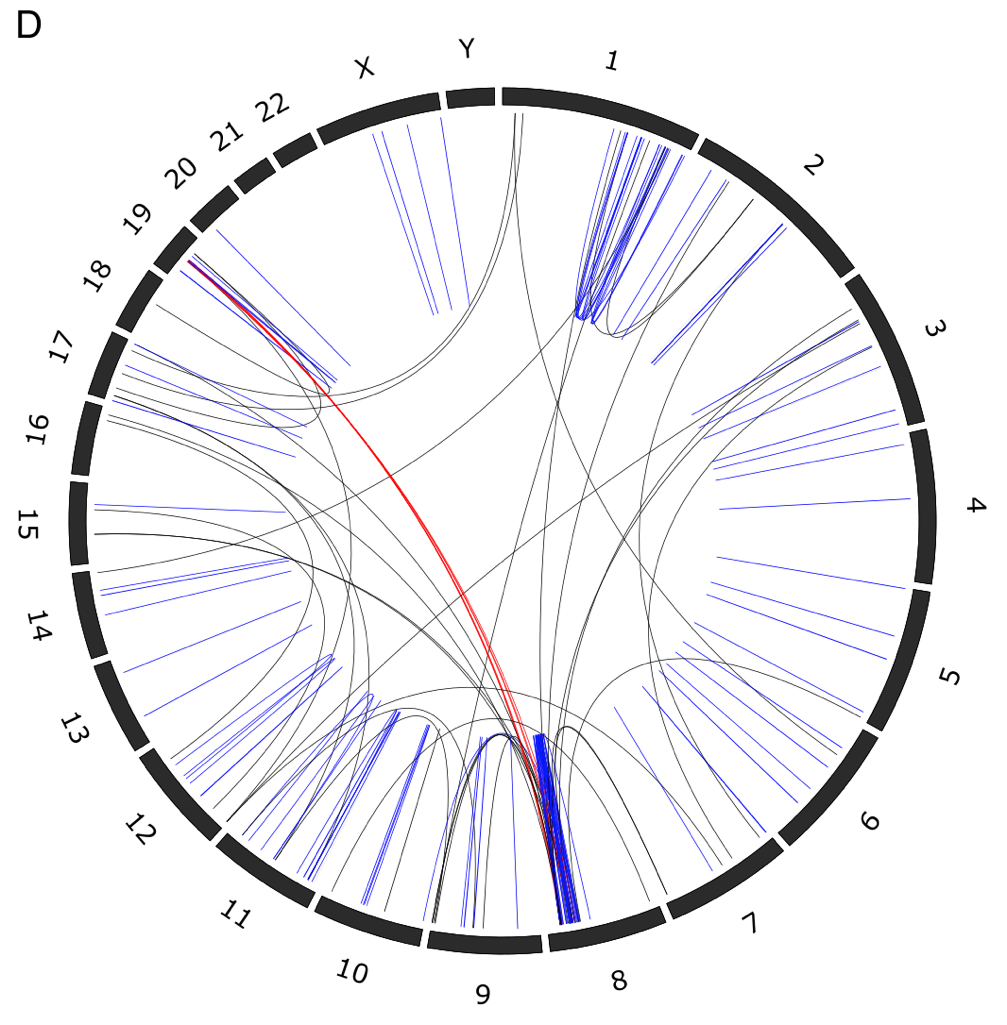

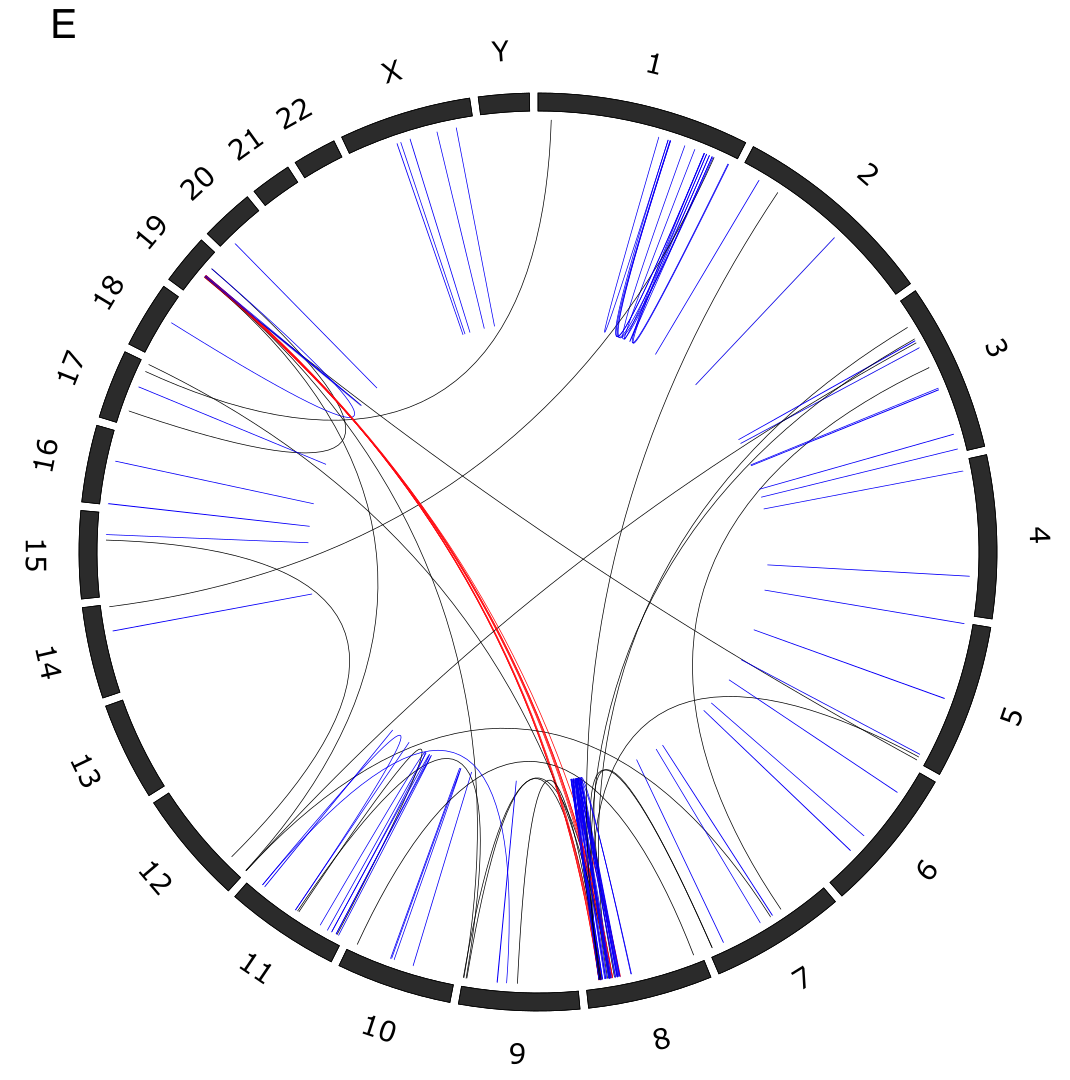


**Figure S2.** Survival analysis showing the impact of (A) *MYC* and (B) *CCNE1* amplifications as detected by FISH in the RNOH cohort (n-= 22). X-axis shows survival time in days.


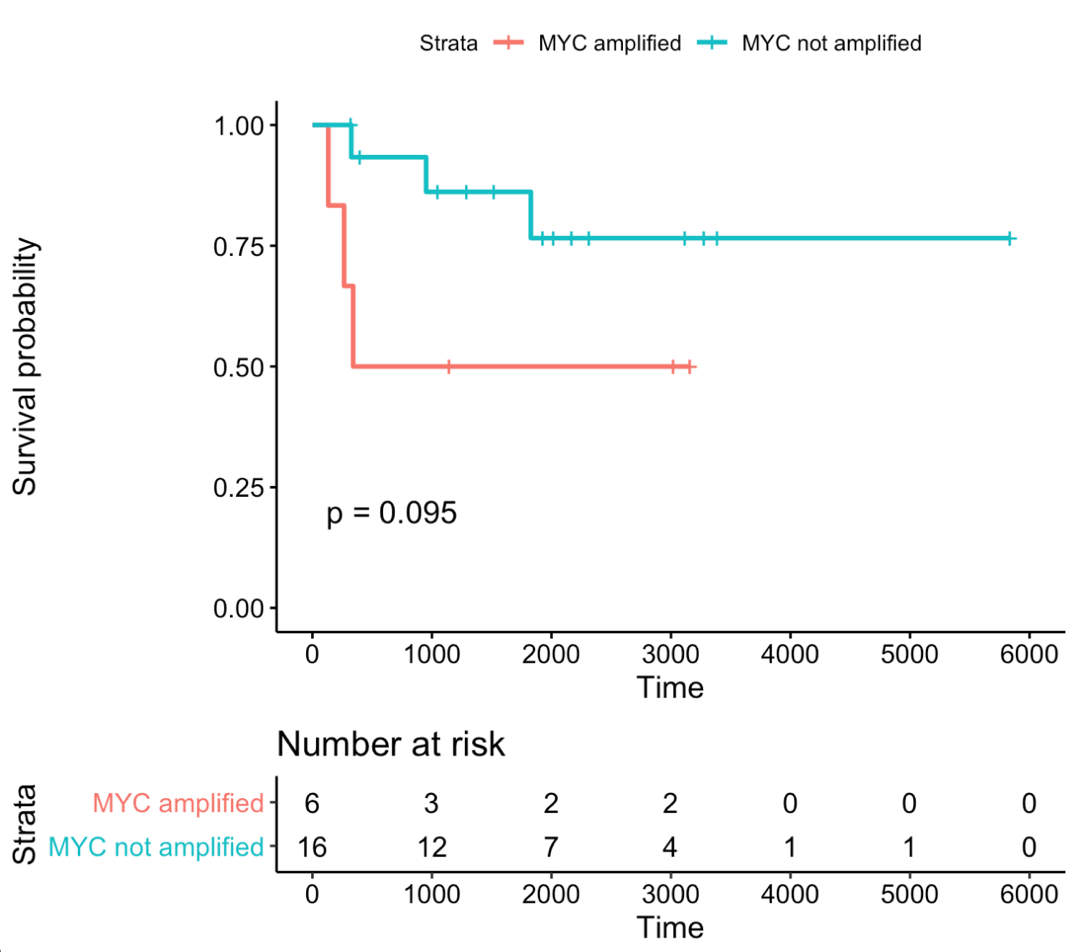
(A)


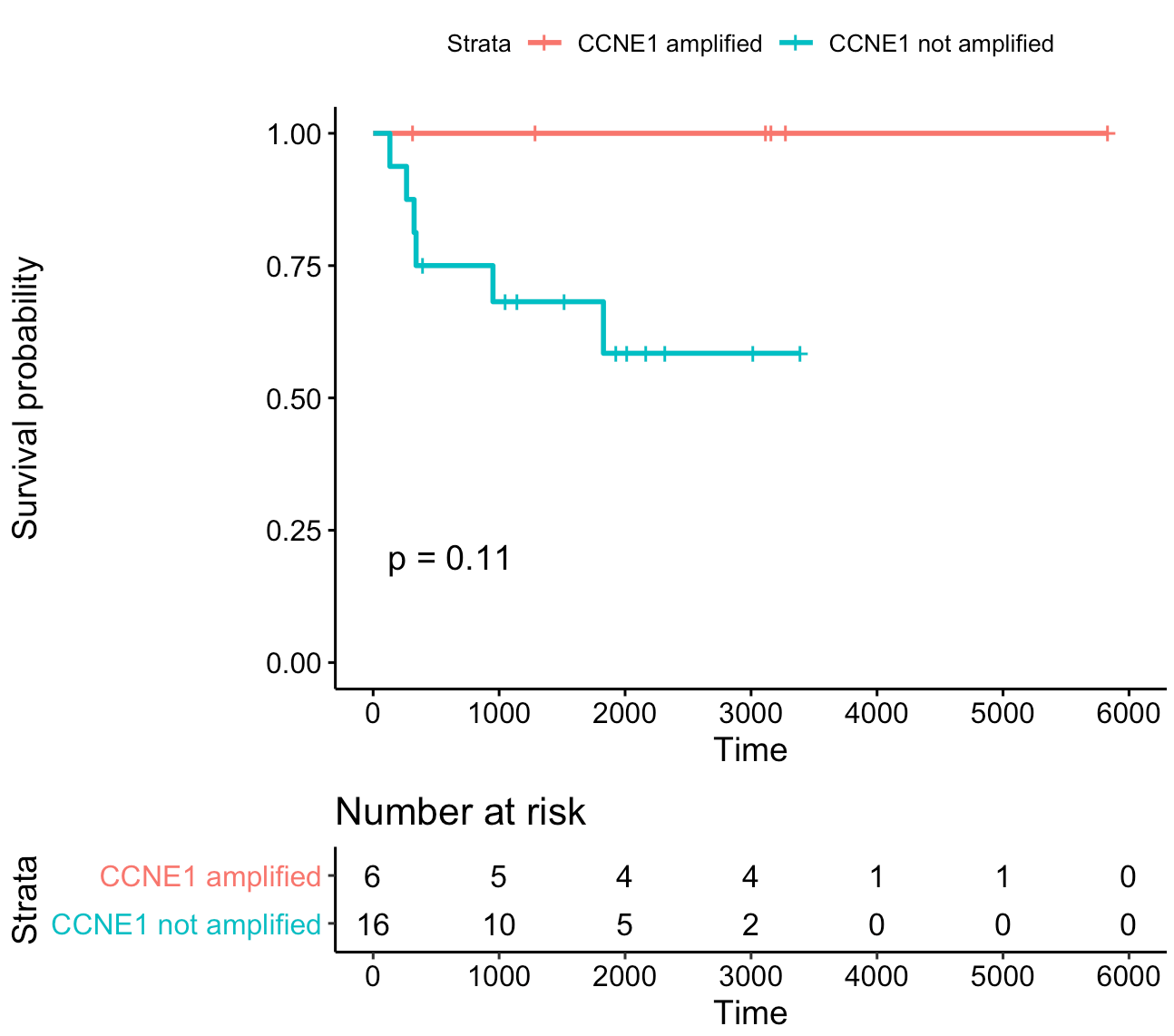


(B)
